# Exploring Prosodic Features Modelling for Secondary Emotions Needed for Empathetic Speech Synthesis

**DOI:** 10.3390/s23062999

**Published:** 2023-03-10

**Authors:** Jesin James, Balamurali B.T., Catherine Watson, Hansjörg Mixdorff

**Affiliations:** 1 Department of Electrical, Computer, and Software Engineering, The University of Auckland, Auckland 1010, New Zealand; 2 Science, Maths and Technology, Singapore University of Technology and Design, Singapore 487372, Singapore; 3 Computer Science and Media, Berliner Hochschule für Technik, 13353 Berlin, Germany

**Keywords:** secondary emotions, emotional speech synthesis, fundamental frequency contour, Fujisaki model, low resource, empathetic speech

## Abstract

A low-resource emotional speech synthesis system for empathetic speech synthesis based on modelling prosody features is presented here. Secondary emotions, identified to be needed for empathetic speech, are modelled and synthesised in this investigation. As secondary emotions are subtle in nature, they are difficult to model compared to primary emotions. This study is one of the few to model secondary emotions in speech as they have not been extensively studied so far. Current speech synthesis research uses large databases and deep learning techniques to develop emotion models. There are many secondary emotions, and hence, developing large databases for each of the secondary emotions is expensive. Hence, this research presents a proof of concept using handcrafted feature extraction and modelling of these features using a low-resource-intensive machine learning approach, thus creating synthetic speech with secondary emotions. Here, a quantitative-model-based transformation is used to shape the emotional speech’s fundamental frequency contour. Speech rate and mean intensity are modelled via rule-based approaches. Using these models, an emotional text-to-speech synthesis system to synthesise five secondary emotions-anxious, apologetic, confident, enthusiastic and worried-is developed. A perception test to evaluate the synthesised emotional speech is also conducted. The participants could identify the correct emotion in a forced response test with a hit rate greater than 65%.

## 1. Introduction

Text-to-speech (TTS) synthesis is used extensively for human-computer interaction. In human-computer interaction, the synthetic speech produced by computer systems (such as conversation agents and robots) is modelled to be humanlike. This humanness in the voice makes the technology more acceptable to users [1,2,3]. In this context, synthesising emotions as produced by humans in social situations is essential. Emotions are broadly classified into primary and secondary emotions. Primary emotions are innate to support fast and reactive response, e.g., *angry*, *happy* and *sad*. Six basic emotions were defined by Ekman [4] based on cross-cultural studies, and the basic emotions were found to be expressed similarly across cultures. The terms ‘primary’ and ‘basic’ emotions are used in the literature with no clear distinction defined between them. For this study, the definition of primary emotions as defined above was used to be in alignment with studies in emotional speech synthesis [5]. Secondary emotions are assumed to arise from higher cognitive processes based on evaluating preferences over outcomes and expectations, e.g., relief and hope [6]. This distinction between the two emotion classes is based on neurobiological research by Damasio [7]. There has been extensive research on primary emotions and methods to synthesise them (a detailed review of past research is provided in Section 2). The studies reported in [2,3] show that for the voice of a robot to be perceived as empathetic, not only primary emotions but secondary emotions are also essential. However, one can expect that modelling secondary emotion is harder compared to modelling primary emotions. This is because secondary emotions are subtle compared to primary emotions. Moreover, lexical information needs to be supported by the appropriate prosodic component to enable people to correctly perceive secondary emotions [8], i.e., the sentence for which emotional speech is synthesised has to be correctly modelled at the accent and phrase levels in alignment with what is being said.

Although there are many secondary emotions, the focus of this study was only on secondary emotions that are needed for human-computer interaction, especially the ones that have been identified to be needed for an empathetic voice. This choice was based on studies on healthcare robots [2,3]. These studies analysed dialogues spoken by the healthcare robot during various scenarios such as greeting the user, providing medicine reminders and guiding the user in tasks. This analysis was followed by a perception test, which suggested that human users perceived empathy in the voice that had secondary emotions. The secondary emotions identified based on the analysis and perception tests in the previously mentioned studies were: *anxious*, *apologetic*, *confident*, *enthusiastic* and *worried*. The same secondary emotions were modelled in this study. Studies on these specific secondary emotions are limited, and so are the number of databases available to analyse them. Therefore, rather than relying on large databases and deep-learning models built based on them, we focused on understanding the impact of the secondary emotions on prosody features-specifically, the fundamental frequency ($f_{0}$) contour. Handcrafted-feature extraction was used to extract $f_{0}$ contour features. The features were then modelled to produce $f_{0}$ contours of secondary emotions. Two other prosody features, namely, speech rate and mean intensity, were modelled by rule-based methods. Modelling the secondary emotions by handcrafted feature extraction on a relatively small database, as described in this paper, leads to developing a low-resource emotional speech synthesis system.

## 2. Past Studies on Emotional Speech Synthesis

A survey of studies focusing on emotional speech synthesis using various techniques from the 1990s is summarised in Table 1. Only the most cited papers that provided a good understanding of the emotional speech synthesis techniques used during these years were reviewed here. The most cited paper before the 2000s [9] had more than 200 citations. Between 2000 to 2010, the most cited paper [10] also had more than 200 citations. Finally, the most cited paper [11] after 2010 also had more than 200 citations, indicating an increased interest in emotional speech synthesis in recent years. This section will help the readers understand the change in requirements for databases and resources over the years for emotional speech synthesis. In the review that follows, a comparison of the size of the speech database needed for each of the approaches is described. The comparison of the database size was made with the databases used in the latest studies (after 2010) as the reference. Studies after 2010 used databases with more than 100 h of recordings [11,12], and these were considered “large” databases. The databases that only had recordings of all diphones of a language [13] were considered relatively small databases, and others that contained a larger number of recordings were considered medium-sized databases.

Studies in the 1990s used rule-based emotional speech synthesis [9,13] on a base voice that was developed using formant/diphone synthesis. (A “base” speech synthesis system refers to the synthesis system that is built initially, which has no emotional modelling. The emotion-based modelling is built on this “base” speech synthesis system.) Diphone synthesis required recordings of all diphones in a language. Formant synthesis modelled the human acoustic system without requiring a large database. Prosody features such as fundamental frequency ($f_{0}$), duration and intensity were modelled by rule-based approaches. The emotion-based rules were derived by extracting these features from a small database for each emotion. The feature extraction used handcrafted approaches, and the changes in features could be explained in terms of the change in emotions.

**Table 1:** Selected emotional speech synthesis techniques from the 1990s.

Speech Synthesis Method	Emotional Speech Synthesis Method	Features Modelled	Resources Needed	Naturalness	Emotions Modelled
1993 [13] Diphone synthesis	Rule-based	Duration and $f_{0}$	All possible diphones in a language have to be recorded for *neutral* TTS ${}^{1}$, e.g., 2431 diphones in British English. An emotional speech database (312 sentences) to frame rules is needed	Average ${}^{2}$	*Neutral*, *joy*, *boredom*, *anger*, *sadness*, *fear* and *indignation*
1995 [9] Formant synthesis	Rule-based	Prosody features such as pitch, duration, voice quality features	DECtalk synthesiser was used containing approximately 160,000 lines of C code. Emotion rules framed from past research	Average	*Anger*, *happiness*, *sadness*, *fear*, *disgust* and *grief*
2004 [14] Parametric speech synthesis	Style control vector associated with the target style transforms the mean vectors of the *neutral* HMM models	$f_{0}$, mel cepstrum	504 phonetically balanced sentences for average voice, and at least 10 sentences of each of the styles	Good	Three styles: *rough*, *joyful* and *sad*
2006 [10] Recorded *neutral* speech used as it is	Rule-based using GMM ${}^{3}$ and CART ${}^{4}$	$f_{0}$, duration	Corpus with 1500 sentences	Average	*Neutral*, *happiness*, *sadness*, *fear* and *anger*
2006 [15] Parametric speech synthesis	Corpus-based using decision trees	$f_{0}$ contours, timing	11 h (excluding silence) of *neutral* sentences + 1 h emotional speech	Good ${}^{5}$	Conveying bad news, yes-no questions
2007 [16] Parametric speech synthesis	Model adaptation on average voice	Mel cepstrum, log $f_{0}$	503 phonetically balanced sentences for average voice, and at least 10 sentences of a particular style	Good	Speaking styles of speakers in the database
2010 [17] *Neutral* voice not created	HMM-based parametric speech synthesis	Spectral envelope, $f_{0}$, duration	Spanish expressive voices corpus-100 min per emotion	Good	*Happiness*, *sadness*, *anger*, *surprise*, *fear* and *disgust*
2017 [12] Parametric speech synthesis using recurrent neural networks with long short-term memory units	Emotion-dependent modelling and unified modelling with emotion codes	Spectrogram	5.5 h emotional speech data + speaker-independent model from 100 h speech data	Reported to be better than HMM-based synthesis	*Neutral*, *happiness*, *anger* and *sadness*
2018 [11] Tacotron-based end-to-end synthesis using DNN ${}^{6}$ (Deep neural network-Tacotron model learning a latent embedding space)	Prosody transfer	Spectrogram	English dataset of audiobook recordings-147 h	Reported to be better than HMM-based synthesis	Speaking styles of speakers in the database
2019 [18] Deep Convolutional TTS	Emotion adaptation from *neutral* TTS to emotional TTS	Spectrogram	Large dataset (24 h) of *neutral* speech + 7000 emotional speech sentences (5 emotions)	Reported to be better than HMM-based synthesis	*Anger*, *happiness*, *sadness* and *neutral*

^1^ Text-to-speech. ^2^ Average and good are used here compared to the methods that were developed in the following years. Average and good do not have any physical significance and are not measurable; rather, they are in comparison to the other methods. ^3^ Gaussian mixed model. ^4^ Classification and regression tree. ^5^ Reduced due to oversmoothing of spectral and excitation parameters by HMM models. ^6^ Tones and break index.

In the early 2000s, the trend shifted to parametric speech synthesis, with hidden Markov model (HMM)-based synthesis being the most popular (see rows three to eight of Table 1). Parametric speech synthesis increased the need for good quality databases (the term good quality here refers to recordings in recording studio environments that have controlled noise levels) with adequate phonetic coverage (between 500 [15,16] to 1500 [10] sentences and larger corpora with 11 h of *neutral* speech recording [15]). (*Neutral* this context refers to speech without any emotions.) Emotions were imparted to the synthesised speech using rules [10], where the rules were derived from a small corpus of each emotion. Corpus-based modelling [15] was also performed, where an emotional speech corpus (one-hour recording for each emotion) was used to derive models for each emotion’s prosody features. Another approach modelled emotional prosody phonology using tones and break index (ToBI)-based $f_{0}$ contour modelling [15]. A one-hour recording for each emotion and 11 h of *neutral* speech recording were used. Another approach was style adaptation using HMM-based synthesis. Style control vectors [14] and the adaptation of acoustic features such as the mel cepstrum and log $f_{0}$ were used. These adaptation methods needed a relatively large database (approximately 500 phonetically balanced sentences [14,16]) to produce an average voice and a smaller database-approximately 10 sentences for each emotion/style-to be adapted [14,16]). All these approaches used the HMM-based synthesis to produce a *neutral* voice and some form of emotion modelling to incorporate emotions onto the *neutral* voice. This required a medium-sized (medium in comparison to the databases needed for the deep-learning approaches explained in the next paragraph) database of *neutral* speech, and a small database of emotional speech to learn from. The features modelled using these approaches were interpretable. However, the naturalness of these synthesised voices was reported to be inferior due to the inherent disadvantage of HMM-based synthesis, that it oversmoothens the spectral and excitation parameters [19]. If a large database for each emotion (100 min of recording per emotion) is available, an HMM-based synthesis can be achieved by training the models based on each of the emotional databases [17] without the need for a *neutral* voice. Such modelling of individual emotions often produces emotional speech with a good naturalness. However, developing large databases for each emotion includes too much overhead, such as the additional requirement to produce recordings for each emotion separately.

After 2015, the trend in speech synthesis shifted towards incorporating neural networks in parametric speech synthesis (see Table 1, rows 9–11). Earlier approaches focused on using recurrent neural networks with long short-term memory units [12]. Then, emotion-dependent modelling was done by inputting emotion code vectors to all model layers based on an emotional speech database. The *neutral* voice was trained using a large database of 100 h of speech, and the emotional speech data were 5.5 h long. The speech produced by such deep neural networks was more natural than with the HMM-based approach, as the oversmoothing of spectral and excitation parameters was avoided. With the improvements in neural networks, the availability of large databases and increased processing power, there has been a lot of focus on developing end-to-end emotional text-to-speech synthesis systems. The Tacotron-based end-to-end speech synthesis system is one of the latest speech synthesis techniques. The study reported in [11] used Tacotron and implemented prosody transfer for emotional speech synthesis. Another research study [18] used a deep convolutional neural network TTS and performed emotion adaptation via transfer learning. Both these neural-network-based approaches required large databases (147 h [11], 24 h *neutral* speech + 7000 emotional speech sentences [18]). The interpretation of the features learned by the neural network was not directly possible. Rather, the learning was based on spectrograms and image-related features, and these features could not be easily associated with the acoustic correlates of speech production.

The last column of Table 1 lists the emotions that have been synthesised by these aforementioned approaches. Most of the emotions synthesised are primary emotions such as angry, sad, happy, fear (in studies [10,17,18,20]) and others focusing on speaking styles (such as studies in [11,14,16]). Only a few studies [13] have synthesised some secondary emotions.

### Emotional Speech Corpus

**Figure 1:**
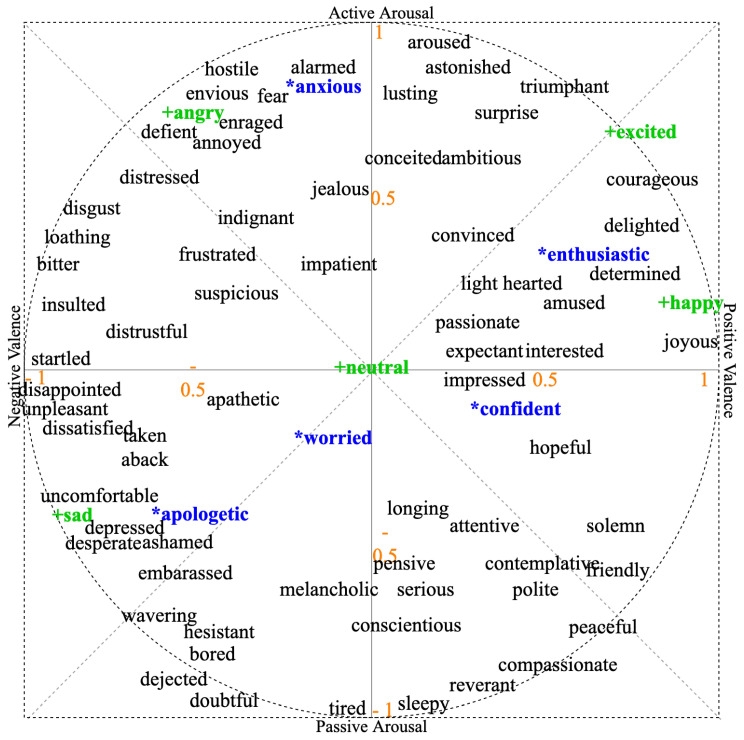
Emotions in the JLCorpus (blue * are the secondary emotions, and green + are the primary emotions) positions on the valence-arousal plane.

The JLCorpus https://www.kaggle.com/tli725/jl-corpus, (accessed on 1 January 2022) [21] contains a total of 2400 emotional speech sentences from 10 emotions (5 primary emotions-*angry*, *excited*, *happy*, *neutral* and *sad*; and 5 secondary emotions-*anxious*, *apologetic*, *confident*, *enthusiastic* and *worried*) spoken by two male (male1 and male2) and two female (female1 and female2) speakers of New Zealand English. The emotions in the JLCorpus are represented on a valence-arousal plane is shown in Figure 1. The valence indicates the pleasantness of the voice ranging from unpleasant (e.g., *sad*, *fear*) to pleasant (e.g., *happy*, *hopeful*). The arousal specifies the reaction level to stimuli ranging from inactive (e.g., *sleepy*, *sad*) to active (e.g., *anger*, *surprise*). Russel developed this model in a psychology study where Canadian participants categorised English stimulus words portraying moods, feelings, affect or emotions. Later, 80 more emotion words were superimposed on Russel’s model based on studies in German [22]. Russel’s circumplex model diagram, as used in this study and shown in Figure 1, was adapted from [23], which was adapted from Russel and Scherer’s work, but the positive valence was depicted by the right side of the x-axis (in contrast to Scherer’s study where it was on the left side.). A two-dimensional model was used (and not higher-dimension models) as representing the emotions on a plane facilitates their visualisation. The JLCorpus is used for this study.

With the motivation to synthesise secondary emotions using a low-resource approach and handcrafted features, the research questions in this investigation were:

*Research question 1:* Can prosody features be used to model secondary emotions?
*Research question 2:* How can a low-resource emotional text-to-speech synthesis system be developed for secondary emotions?

## 3. Emotional Speech Corpus Analysis

Even though the JLCorpus contains both primary and secondary emotions, the focus of this research was only on secondary emotions. The primary emotions *sad* and *excited* were added in the plots to represent the extremities in the valence-arousal levels, and a *neutral* emotion was added as a baseline for comparison. This will help the reader understand the relative behaviour of the secondary emotions compared to the primary emotions. Many features were used to represent speech and audio signals. Examples of features included mel frequency cepstral coefficients (MFCCs); spectral features such as jitter and shimmer [24,25]; glottal features such as the open quotient and closed quotient; and prosody features such as the fundamental frequency ($f_{0}$), speech rate and mean intensity. The analysis presented focused on three prosody features: the fundamental frequency ($f_{0}$), speech rate and mean intensity. These three features were considered as they have been extensively used in past research (examples can be found in Table 1) for emotional speech synthesis. Only the results of male2 and female2 speakers are discussed as they had the highest perception accuracy among all four speakers from the perception test for evaluating the JLCorpus [21]. Averaging the results across all the speakers would cause these feature values to not have distinct emotion-dependent regions. Therefore, speaker-based results averaged across the sentences per speaker per emotion are presented here. There were 60 sentences per speaker for every emotion; in total, the results corresponded to 960 sentences. When the valence-arousal levels are described to relate to change in prosody features, the two-dimensional space shown in Figure 1 was used as the reference.

### 3.1. Prosody Feature Analysis

**Figure 2:**
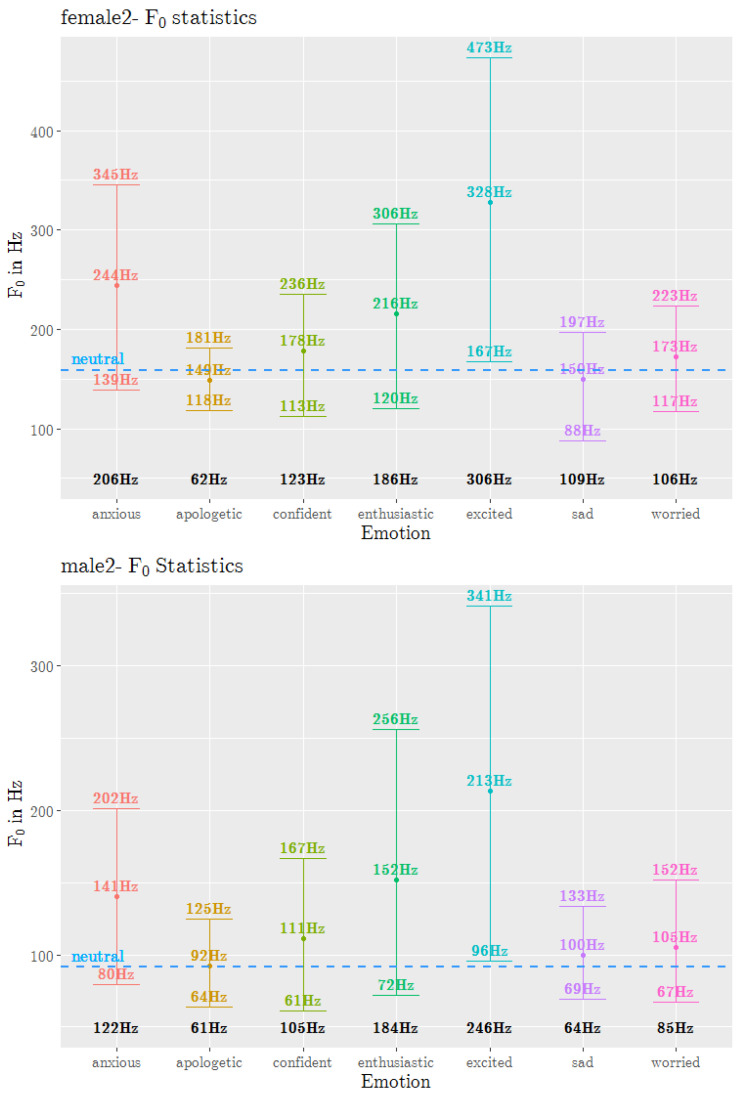
Fundamental frequency statistics of 5 secondary emotions, and *sad*, *excited* and *neutral* from the JLCorpus for speakers female2 and male2. The dot on each line represents the mean $f_{0}$, and the upper and lower bounds indicate the maximum and minimum values, respectively. The bold black number at the bottom is the $f_{0}$ range. The dash lines represent the results for *neutral*.

The $f_{0}$ track was extracted from the JLCorpus using the *wrassp* wrapper [26], an advanced speech signal processor library in R computing software [27]. The ksvF0 fundamental frequency estimation function was used with its default settings. The $f_{0}$ track was averaged at the sentence level to obtain the mean $f_{0}$. The minimum, maximum and range of $f_{0}$ were calculated for every sentence. These were then averaged across all sentences to obtain the $f_{0}$ statistics for *sad, excited and neutral* and five secondary emotions, and this result is shown in Figure 2. The dot represents the mean $f_{0}$, and the upper and lower bounds indicate the maximum and minimum values, respectively. The bold black number at the bottom of the graph is the $f_{0}$ range. The plotting was done using R’s ggplot [28]. Even though the frequency range for female2 and male2 speakers were different, the effect of emotions on $f_{0}$ had common trends. Among the secondary emotions, *enthusiastic* and *anxious* had the highest mean $f_{0}$ (high arousal emotions), with *enthusiastic* having the largest range (averaged across male2 and female2 speakers). *Apologetic* (low arousal emotion) had the lowest mean $f_{0}$ and range. *Confident* and *worried* fell in between the other three emotions that had more extreme values. Even though emotions such as *apologetic*, *confident* and *worried* had similar mean $f_{0}$ values, the $f_{0}$ range differentiated them. *Confident* in the fourth quadrant of the valence-arousal plane had a mean $f_{0}$ similar to *neutral* and *worried*; however, its range was higher than that of *worried*, which may have been an effect of its slightly higher arousal level or positive valence level. Interestingly, the primary emotion *sad* did not have the lowest mean $f_{0}$ value despite having the lowest valence level. A more detailed analysis of the fundamental frequency contour of secondary emotions was conducted by the authors and is reported in [29].

**Figure 3:**
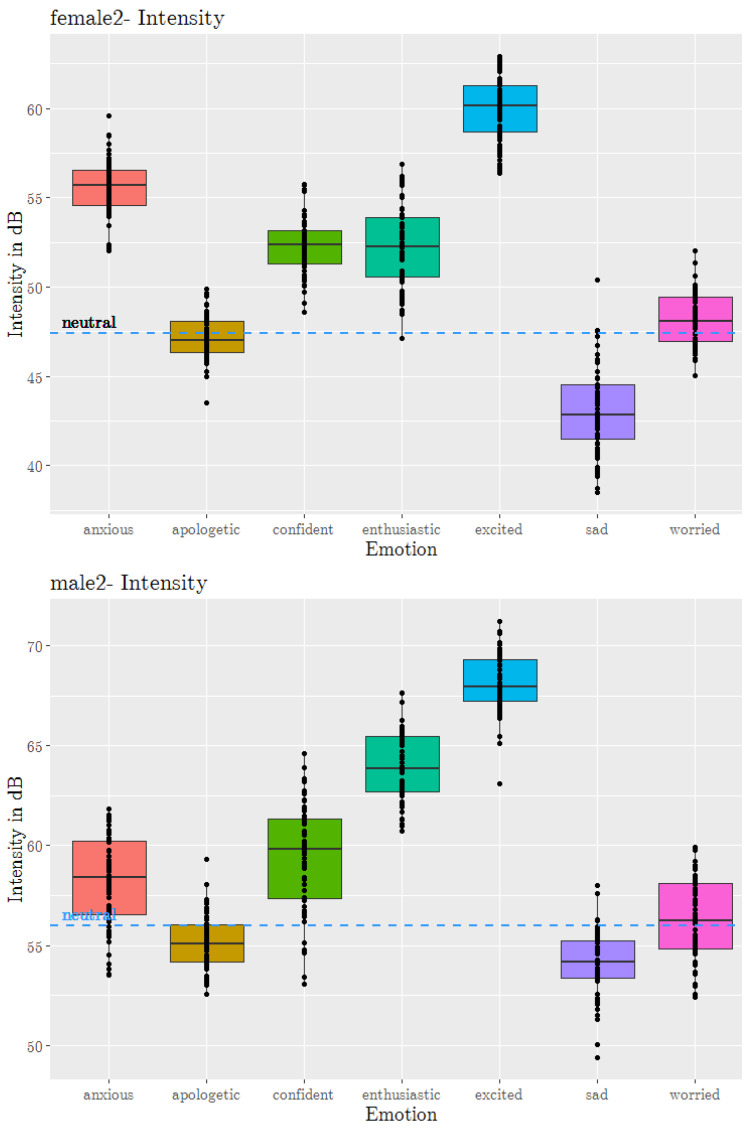
Mean intensity boxplots of 5 secondary emotions, and *sad*, *excited* and *neutral* from the JLCorpus for speakers female2 and male2.

The mean intensity in decibels (dB) was measured using the *wrassp* wrapper using the rmsana short-term root-mean-square amplitude analysis function with default settings. The intensity was averaged at the sentence level. Figure 3 shows boxplots for the intensity across *sad, excited* and *neutral*, and 5 secondary emotions plotted using R’s ggplot. Each point on the plot represents the mean intensity of a sentence. The dashed line is the mean intensity for *neutral*. It can be seen that emotions influenced the intensity very strongly. Distinct regions, often with little or no overlap, can be seen in the boxplots. In contrast to the $f_{0}$ values, the mean intensity values for the primary emotions *excited* and *sad* were clearly at the extremities. Among the secondary emotions, *enthusiastic* and *anxious* (high arousal) had the highest intensity, and *worried* and *apologetic* (low arousal) had the lowest. All high-arousal emotions (*anxious*, *enthusiastic*) were much above the *neutral* line and low-arousal emotions (*apologetic*, *worried*) were near or below it. *Confident* with arousal levels near *neutral* had intensity values slightly higher than *neutral*. This could be due to the positive valence of *confident*, and this claim needs to be investigated further. Moreover, *confident* had a slightly higher arousal than *worried*, resulting in higher mean intensity values.

**Figure 4:**
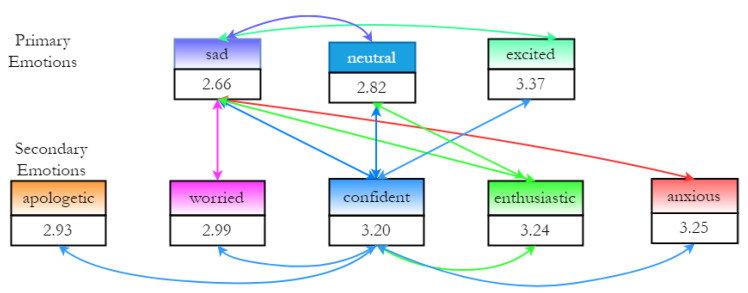
Speech rate statistics of 5 secondary emotions, and *sad*, *excited* and *neutral* from the JLCorpus for speakers female2 and male2.

Finally, the speech rate in syllables per second was calculated by counting the number of syllables per sentence, and it was divided by the sentence duration [30]. The speech rate variations were not as pronounced as those of $f_{0}$ and the intensity due to the short duration of the sentences in the JLCorpus (as noted in [21]). An additional statistical analysis was conducted to understand the effect of emotions on speech rate. Figure 4 shows the statistical analysis results (from R) for eight emotions, with the value in the box representing the average speech rate for each emotion, where significantly different emotion pairs obtained from a pairwise *t*-test are marked by arrows. The emotion with the lowest average speech rate was the primary emotion *sad* (low arousal), and it was significantly different from all other emotions except *apologetic*, perhaps expectedly due to their similar valence and arousal levels. The primary emotion *excited* (high arousal) had the highest average speech rate, followed by *anxious* and *enthusiastic*. *Confident* showed a significant difference in speech rate from all other emotions. This could be a result of its unique position in the fourth quadrant (see Figure 1). Overall, the results suggested that with reducing levels of arousal from *enthusiastic* to *apologetic*, the speech rate reduced.

To summarise, for high arousal emotions (such as *anxious, enthusiastic and confident*), the feature values for $f_{0}$ and the mean intensity were high. For low arousal emotions (such as *apologetic, sad and worried*), the feature values were low. For the speech rate, all emotions followed an increasing trend of feature values as the arousal level of the emotions increased. Thus, all three prosody features were arousal-differentiating. It was found that *confident* behaved similarly to *worried* for the arousal-differentiating features. The results of this analysis suggested that the three prosody features were impacted by secondary emotions. Hence, modelling these three prosody features could be effective in synthesising these secondary emotions.

### 3.2. $f_{0}$ Contour Analysis

**Figure 5:**
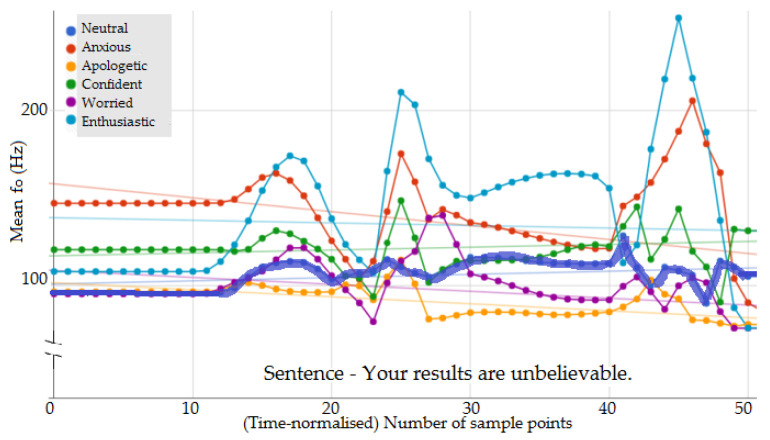
$f_{0}$ contour of 5 secondary emotions and *neutral* (dark blue bold line).

Figure 5 shows the time-normalised $f_{0}$ contour for five secondary emotions and *neutral* extracted for the same sentence. Comparing *neutral* and secondary emotions, there were clear differences in the mean and range of $f_{0}$ (also noted in the statistical analysis reported in Section 3.1); most importantly, the $f_{0}$ contour shapes showed considerable differences. For example, in Figure 5, consider the sample points between 10 to 20. One can see that the shape of the contour for *apologetic* is visually different from that of *enthusiastic*, and it is not just a difference in the mean and range values alone; it is in the timing of the peak of the contour. This indicated that even though a qualitative model based on the $f_{0}$ statistics could provide emotion separation for primary emotions [5], such a model may be insufficient to capture the $f_{0}$ contour variations of secondary emotions.

Contour-based modelling conveys concurrent linguistic information (e.g., sentence modality, word prominence) and paralinguistic information such as emotions [29]. The Fujisaki model is one of the classic $f_{0}$ contour models [31]. Fujisaki’s model approximates the natural $f_{0}$ contour and interpolates through unvoiced sounds. The model is event-based, i.e., every command is related to the onset of a new phrase, accented syllable or boundary tone. This model quantifies the $f_{0}$ contour with a few parameters from which the $f_{0}$ contour can be constructed. Studies reported in [32,33,34,35] used the Fujisaki model for various speech signal applications.

**Figure 6:**
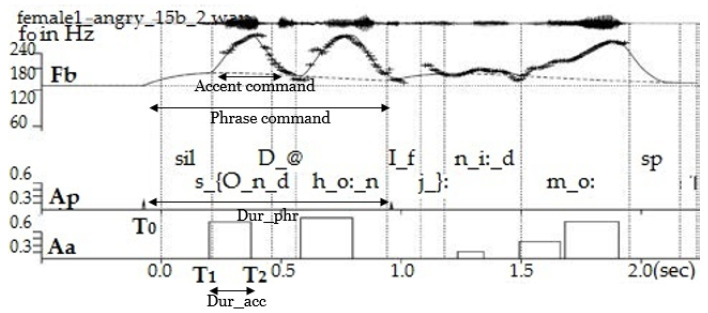
Fujisaki parameters for *“Sound the horn if you need more”* (SAMPA symbols). $T_{0}$, $T_{1}$ and $T_{2}$ marked for the first phrase and accent commands only.

The Fujisaki model [35] parameterises the $f_{0}$ contour superimposing (see Figure 6) (1) the base frequency $F_{b}$ (indicated by the horizontal line at the floor of the $f_{0}$ pattern), (2) the phrase component-declining phrasal contours accompanying each prosodic phrase-and (3) the accent component-reflecting fast $f_{0}$ movements on accented syllables and boundary tones. These components are specified by the following parameters:

1. *Phrase command onset time ($T_{0}$)*: Onset time of the phrasal contour, typically before the segmental onset of the phrase of the ensuing prosodic phrase. Phrase command duration *Dur_phr*= *End of phrase time*-$T_{0}$.
2. *Phrase command amplitude ($A_{p}$)*: magnitude of the phrase command that precedes each new prosodic phrase, quantifying the reset of the declining phrase component.
3. *Accent command amplitude ($A_{a}$)*: amplitude of the accent command associated with every pitch accent.
4. *Accent command onset time ($T_{1}$) and offset time ($T_{2}$)*: The timing of the accent command can be related to the timing of the underlying segments. Accent command duration *Dur_acc* = $T_{2}$−$T_{1}$.

Using the parameters, the $f_{0}$ contour can be obtained as:

(1) $$\begin{array}{c} ln\left(f_{0}\left(t\right)\right)=ln\left(F_{b}\right)+\sum_{i=1}^{I}A_{pi}G_{pi}\left(t-T_{0i}\right)+\\ \sum_{j=1}^{J}A_{aj}\left(G_{aj}\left(t-T_{1j}\right)-G_{aj}\left(t-T_{2j}\right)\right) \end{array}$$

where

(2) $$\begin{array}{c} G_{pi}\left(t\right)=\left\{\begin{array}{cc} \alpha_{i}^{2}texp\left(-\alpha_{i}t\right), & \forall t\geq 0.\\ 0, & \mathrm{otherwise}. \end{array}\right. \end{array}$$

(3) $$\begin{array}{c} G_{aj}\left(t\right)=\left\{\begin{array}{cc} min[\gamma_{j},1-\left(1+\beta_{j}t\right)exp\left(-\beta_{j}t\right)], & \forall t\geq 0.\\ 0, & \mathrm{otherwise}. \end{array}\right. \end{array}$$

$F_{b}$-Bias level upon which all the phrase and accent components are superposed to form an $f_{0}$ contour.
$\beta_{j}$-Natural angular frequency of the *j*th accent command.
$\alpha_{j}$-Natural angular frequency of the *j*th phrase command.
*I*-Number of phrase commands.
*J*-Number of accent commands.
$A_{pi}$-Magnitude of the *i*th phrase command.
$A_{aj}$-Magnitude of the *j*th accent command.
$T_{0i}$-Instant of occurrence of the *i*th phrase command.
$T_{1j}$-Onset of the *j*th accent command.
$T_{2j}$-Offset of the *j*th accent command.
$\gamma_{j}$-Ceiling level of the accent component for the *j*th accent command.

$A_{a}$, $A_{p}$, $T_{0}$, $T_{1}$, $T_{2}$, $F_{b}$, α, β and γ are referred to as the Fujisaki parameters. In this study, α and β were kept constant, and the other six parameters were modelled for the different emotions.

#### 3.2.1. Fujisaki Parameterisation of $f_{0}$ Contour

The $f_{0}$ contour was extracted with the Praat standard method [36] for every 0.01 s. Then, the Fujisaki model parameters were estimated from the natural $f_{0}$ contour using an automatic algorithm called the AutoFuji extractor [37]. In the analysis of reading-style speech, typically, every content word is characterised by at least one accent command associated with the primary pitch accent, and the base frequency $F_{b}$ is kept constant for each speaker [38]. In the context of emotional speech, however, in principle, every syllable can exhibit an accent command, especially when the emotion entails a strong arousal. Sometimes even a single syllable that is strongly emphasised can contain two accent commands as seen in the syllable “m_o:” of Figure 6 (see between time 1.5 to 2 s). The Fujisaki model parameters for each utterance were checked to ensure that potential errors in $f_{0}$ tracking did not tamper the parameter, leading to additional accent commands in unvoiced segments. This hand- checking was done by the first and fourth authors of this paper, with the first author checking all the files once and correcting them, followed by the fourth author rechecking them. In most cases, the $F_{b}$ set for the automatic algorithm was used as is. However, for certain speakers, the $F_{b}$ had to be adjusted (±10 Hz) as a function of the emotion portrayed to make the Fujisaki estimated contour better fit the original $f_{0}$ contour. This process could not be conducted automatically because errors in $f_{0}$ tracking could cause wrong Fujisaki parameter estimations.

There were 2400 short utterances in the JLCorpus, out of which 1200 were analysed. (Both male and female speakers’ sentences were used for the initial analysis. However, only the male speaker sentences were used for the emotion-based $f_{0}$ contour model developed because it was that male speaker’s voice that was synthesised). Only a subset of the corpus was analysed because the Fujisaki parameterisation required hand-correction. Moreover, by taking a subset, two renderings of each sentence in the JLCorpus (out of four available) for each emotion for each speaker were analysed. As we aimed to synthesise these $f_{0}$ contours, getting an accurate parameterisation of the contour for a given sentence was important. Hence, two renderings of the same sentence were used to capture the $f_{0}$ contour accurately. The impact of adding more renderings of the same sentence on the model will need to be tested in the future when more data get hand-corrected.

Finally, an automatic time alignment of the Fujisaki parameters with each of the syllables in the corpus was performed, i.e., accent commands were associated with the syllables in which they began and ended, and phrase commands with the initial syllables of phrases that they preceded. The results were collated, and it contained the Fujisaki model parameters ($A_{a}$, $A_{p}$, $F_{b}$, $T_{0}$, $T_{1}$, $T_{2}$) for each of the syllables. The analysis of the effect of emotions on the Fujisaki parameters (detailed report in [29]) showed that they were affected by the emotions, with accent command parameters (smaller units-$A_{a}$ and accent command duration $T_{2}-T_{1}$) and $F_{b}$ having the most significant effect.

The analysis in Section 3.1 showed that the secondary emotions could be differentiated using the mean intensity. Hence, a rule-based approach was used to model the mean intensity of the secondary emotions during synthesis. The intensity contour modelling was not attempted here, as past research has not determined a well-established intensity contour model at the accent and phrase level. Hence, developing a new intensity contour model is reserved for future investigation.

A rule-based approach was used to model the speech rate. Modelling the $f_{0}$ contour by the Fujisaki model and mean intensity and speech rate using rules addressed *research question 1.*

## 4. Emotional TTS Synthesis System Development

**Figure 7:**
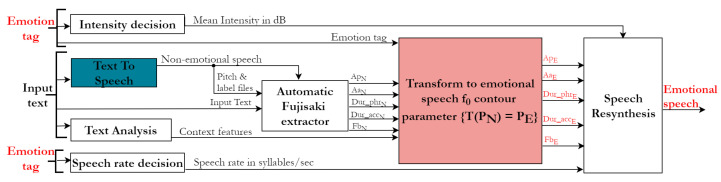
Emotional text-to-speech synthesis system with $f_{0}$ contour transformation.

Here, we address research question 2. The overall system diagram for the TTS synthesis system is shown in Figure 7. The inputs to the system were the text to be converted to speech and the emotion tag to which the conversion had to be done. Synthesised speech was produced from the input text using a text-to-speech module. This produced synthesised speech that had no emotions. The $f_{0}$ contour’s Fujisaki parameters of the nonemotional speech were extracted via the automatic Fujisaki extractor module. The nonemotional $f_{0}$ contour’s Fujisaki parameters were transformed into emotional $f_{0}$ contour parameters. Using these emotional $f_{0}$ contour parameters, the $f_{0}$ contour corresponding to the emotion tag was reconstructed. The intensity and speech rate decisions were made using the emotion tag. Finally, using the reconstructed $f_{0}$ contour, the intensity and speech rate values, emotional speech was resynthesised. Details about each module are given in the following sections.

### 4.1. Text-to-Speech Module

Previous work [39,40] has led to the development of a TTS synthesis system in New Zealand English based on MaryTTS [41] (with approximately 1000 sentences spoken by a male New Zealand English speaker). Synthesised speech for New Zealand English is currently without any emotion and is called *nonemotional speech* here. The input text was passed through the New Zealand English MaryTTS system, and the output nonemotional speech was obtained.

### 4.2. Automatic Fujisaki Extractor

The $f_{0}$ contour was extracted from the nonemotional speech (by the *Praat* Auto Correlation Function method [42]). Label files were obtained from the input text and nonemotional speech using the New Zealand English option of the Munich Automatic Web Segmentation System [43]. The pitch and label files were provided to the AutoFuji extractor [37] to obtain the five derived Fujisaki model parameters of nonemotional speech-$A_{pN}$, $A_{aN}$, $Dur\_phr_{N}$, $Dur\_acc_{N}$, $F_{bN}$, where *N* represents “nonemotional". The parameters were then time-aligned to the text at the phonetic level. These Fujisaki parameters were obtained via an automatic process and were not hand-corrected. The Fujisaki parameters were transformed into corresponding emotional speech parameters. Hence, hand-correction of these nonemotional $f_{0}$ contour parameters was not necessary. Avoiding hand-correction also made real-time synthesis possible, which is suited for human-computer interaction applications.

### 4.3. Transformation to Emotional Speech Parameters

This module transformed the Fujisaki model parameters of the nonemotional speech’s $f_{0}$ contour to that of emotional speech. For conducting this transformation, a regression model was developed, as described here.

#### 4.3.1. Features for the Regression Model

The only inputs available for an emotional TTS synthesis system are the text to be converted to speech and the emotion to which the speech has to be transformed. For a real-time implementation, all the features used for transforming the $f_{0}$ contour parameters here were based on these two inputs only. A list of all features, along with their extraction methods, is given in Table 2. In total, 109 features were extracted.

*Linguistic context features* refer to a set of features that describe the phonetic environment of the target phoneme. In this investigation, the linguistic features used in MaryTTS [41] were used for the $f_{0}$ contour prediction. This choice was further motivated by the fact that MaryTTS was our front-end synthesiser (more details in Section 4.3.2). Examples of context features are the forward/backward position of a phoneme in a syllable, the number of accented/stressed syllables before/after the current syllable, ToBI end-tone marking, etc. This feature extraction process is represented by the *text analysis* module in Figure 7.

The *nonemotional $f_{0}$ contour Fujisaki parameters* were used as features for the transformation. The extraction of these features is represented by the *automatic Fujisaki extractor* module in Figure 7 as described above in Section 4.2.

Another feature used was the *emotion tag* representing the emotion to which the transformation had to be done. The database for training the transformation model contained two male speakers. Hence, a *speaker tag* was also used as a feature for the transformation model development.

#### 4.3.2. $f_{0}$ Contour Transformation Model

The set of hand-corrected Fujisaki parameters (the extraction and hand correction described in Section 3.2.1) obtained from the natural emotional speech in the JLCorpus was the target value to be predicted by the model. There were 7413 phoneme tokens from two male speakers of the JLCorpus. These phoneme tokens and corresponding Fujisaki model parameters formed the database for the $f_{0}$ contour transformation model development. In total, 80% of the database was used for training, and 20% was used for testing using random selection. It was ensured that parts of the same sentences were not split into the training and test set. This was done by choosing three sentences used for testing (which accounted for approximately 20% of the total tokens) and the remaining 12 sentences used for training. (In total, there were 15 different sentences in the JLCorpus, for each emotion, each speaker and each repetition.) As seen in Figure 7, the input to the $f_{0}$ contour transformation model was the nonemotional $f_{0}$ contour features, emotion tag and context features. These were the features based on which the $f_{0}$ contour transformation model was trained. A less resource-intensive machine-learning-based regression model was developed using the handcrafted features extracted here. The transformation predicted Fujisaki model parameters for every phoneme in an input sentence based on the emotion tag to which the conversion needed to be done.

The $f_{0}$ contour transformation model training was as follows.

Here, we chose two ensemble regressors-random forest [44] and Adaboost [45]-as a proof of concept to implement ensemble-learning-based regression. Both random forest and Adaboost were allowed to run independently, and the outputs obtained from the two regressors were aggregated in the end without giving any preference to either of the algorithms by taking the mean of the predictions from both algorithms. The implementation was done in Python using the scikit-learn machine learning library RandomForestClassifier and AdaBoostRegressor packages [46]. Based on 109 features corresponding to each phoneme in the training set, the two regression algorithms were individually trained to learn the patterns of emotional speech’s $f_{0}$ contour parameters. The use of ensemble methods has the important advantages of an increase in accuracy and robustness when compared to the use of a single model [47]. This makes ensemble methods suited for applications where small improvements in the predictions have an important impact. This is relevant here, as the requirement is to predict the Fujisaki model parameters accurately, which are numbers, and small variations in them can cause the parameters to change to that of another emotion. The hyperparameters (for *random forest*, the number of trees, the maximum number of features considered for node splitting, the maximum number of levels in each decision tree, the minimum number of data points placed in a node before the node is split, the minimum number of data points allowed in a leaf node, the method of sampling data points; and for *Adaboost*, the number of estimators, learning rate and number of splits) of these supervised learning methods were tuned via a grid-search cross-validation, and the best parameter set was used for the training. The mean of the predictions from the two algorithms was taken as the final prediction. Such ensemble-learning-based regression models were developed for each emotion. These emotion-dependent models were combined to form the emotion transformation model for the $f_{0}$ contour parameters.

#### 4.3.3. Using the Transformation Model

Let each nonemotional speech’s $f_{0}$ contour parameter be called $P_{N}$, *N* stands for “nonemotional”. Then, the developed transformation model ($T\left(P_{N}\right)=P_{E}$) was applied to this nonemotional speech’s $f_{0}$ contour parameter based on the features. $P_{E}$ denotes emotional speech parameters. The transformed parameters were then used to produce the $f_{0}$ contour of the emotional speech. This step is represented by the *transform to emotional speech $f_{0}$ contour parameter* module in Figure 7.

### 4.4. Speech Rate and Mean Intensity Modelling

Table 3 lists the mean speech rate and mean intensity for each of the five secondary emotions estimated by analysing the JLCorpus (as described in Section 3.1). Based on these intensity and speech rate values, rules were identified for each emotion. These rules were then applied to the speech signal after the $f_{0}$ contour transformation was performed.

### 4.5. Resynthesis

The Fujisaki parameters predicted for each phoneme in a sentence were time-aligned to the sentence’s phonemes. Accent and phrase commands were placed based on this time alignment, and the $F_{b}$ was assigned to the sentence. If the model predicted that the accent/phrase command positions needed to be changed compared to nonemotional speech, then accent/phrase commands were added/deleted/shifted accordingly. The Fujisaki parameters were then used to reconstruct the $f_{0}$ contour by superimposing the $F_{b}$, accent commands and phrase commands. Once the $f_{0}$ contour was reconstructed, emotional speech was resynthesised by pitch-synchronous overlap-and-add using *Praat*.

## 5. Performance Analysis and Results

Resynthesised emotional speech was evaluated by a subjective test with 29 participants out of which 14 participants had English (all variants of English included) as their first language (called L1 speakers). In total, 24 participants were from the age group 16–35, and the remaining were distributed over 36–65. All the participants had average, above-average or excellent (self-reported) hearing. Twenty-three participants used headphones, five used loudspeakers, and the remaining one used a laptop speaker. The survey was designed on Qualtrics, a web-based survey platform. The average time taken by the participants to complete the test was 40 min. The perception test was divided into five tasks to evaluate the various aspects of the synthesised emotional speech. The participants did the entire test in one sitting. The survey automatically proceeded to the next task when one task was completed. The tasks were presented to the participants in the same order as described here. We also interpreted the applicability of these results for a healthcare robot for which this TTS system was developed.

Different aspects of the synthesised emotional speech were evaluated. These included evaluating if participants could identify the emotion in the synthesised speech from a set of emotion names provided, evaluating if participants could identify the emotion in the synthesised speech if no emotion names were provided, evaluating the naturalness of the synthesised speech and the comfort level of listening to the synthesised speech.

### 5.1. Task I-Pairwise Forced-Response Test for Five Secondary Emotions

This task aimed to evaluate if the participants could differentiate five secondary emotions when two of them were presented. Participants listened to 100 sentences and grouped them into the two emotion names provided. These sentences were divided into blocks of 10. Most of the sentences were from the JLCorpus [21]. All five secondary emotions were perceptually evaluated in this pairwise test. This was a forced-response test, as the participants could only choose from the emotion list given to them. The 100 sentences were evaluated by the 29 participants, giving 2900 evaluations. The training was provided to the participants to acquaint them with the type of utterances to expect and give them practice in doing the task.

The results of the test are summarised as a collection of confusion matrices, one for each emotion pair shown in Table 4. The horizontal rows indicate the perceived emotions of the participants, and the vertical columns indicate the actual emotions. Each confusion matrix shows the *perception accuracy in percentage* (calculated by the number of correct choices of the emotion divided by the total number of sentences of that emotion as a percentage) for each of the emotion pairs. The highlighted percentage value in the table represents the cases where the participants perceived the actual emotion correctly. The Kappa statistics, κ=0.816 (95% confidence interval, 0.813 to 0.818, p<0.0001), showed strong inter-rater agreement, which means that there was consistency among the participants in differentiating the emotion pairs. Looking at the results, one can deduce that the most confusing emotion pairs were *enthusiastic* vs. *anxious*, *confident* vs. *enthusiastic* and *apologetic* vs. *worried* (which might be due to their closeness in the valence and arousal levels). Each participant evaluated each emotion pair 10 times, ensuring that each emotion pair was evaluated 290 times. Thus, it was reasonable to assume that the perception accuracy was much higher than chance. Overall, the average perception accuracy across all emotions was 87%. Comparing these results with past studies that modelled the $f_{0}$ contour for emotional speech synthesis such as [48] (reported 50% perception accuracy for expressions good news, bad news and question), Refs. [49,50] (reported 75% perception accuracy for *happy*, *angry* and *neutral*) and [51] (reported 65% perception accuracy for *joy*, *sadness*, *anger* and *fear*), the results obtained here were comparable to past studies. However, past studies did not report $f_{0}$ contour modelling for these secondary emotions. Hence, a direct comparison was not possible.

In the natural speech subjective test conducted previously by the authors [21], the emotions that were difficult to differentiate were *enthusiastic* vs. *anxious*, *confident* vs. *enthusiastic* and *worried* vs. *apologetic*. From the confusion matrices shown in Table 4, it can be seen that the emotions pairs that were most difficult to differentiate were *worried* vs. *apologetic* and *confident* vs. *enthusiastic*, as these pairs had the lowest correct hit rates (i.e., the dialogue elements of the confusion matrix). The emotions that were most easy to differentiate were *apologetic* vs. *anxious* and *apologetic* vs. *enthusiastic*. These observations indicate that synthesising emotions such as *apologetic and worried*may require modelling other acoustic features than the ones considered in this study. From this analysis, it was found that some emotion pairs were easier to differentiate compared to others, and this could be related to the valence-arousal levels of the emotions in the pair. Among the most difficult emotions to differentiate, *apologetic* vs. *worried* and *enthusiastic* vs. *confident* may not be problematic for users of a healthcare robot. This is because, for example, if the healthcare robot is speaking enthusiastically but it is wrongly perceived as confident by the user, it will not negatively impact the user’s perception and reaction to the robot. However, the confusion between *enthusiastic* vs. *anxious* will cause difficulty for the users, as a healthcare robot (or any human-computer interaction application) that speaks enthusiastically but is perceived as anxious by the user would baffle the user. Future work on modelling these emotions will have to concentrate on these two emotion pairs in detail. Moreover, the words in the sentences spoken with these emotions can also help differentiate between *enthusiastic* and *anxious.*

### 5.2. Task II-Free-Response Test for Five Secondary Emotions

In this task, the participants listened to one sentence at a time and wrote down any number of emotions they perceived. This was a free-response test, and the participants were not given any emotion options to choose from. The sentences they heard were a subset of the collection of sentences used for the forced-response tests, and they were different for each emotion. Two sentences corresponding to emotions were evaluated, making a total of 10 sentences evaluated by 29 participants, giving 290 evaluations.

The emotion words written by the participants for each of the five secondary emotions, along with the number of times each word was written, is given in Table 5. It can be seen that the free responses entered by the participants were almost in alignment with the intended emotion. A major confusion was for actual emotion *enthusiastic*, which was reported as *confident* by many participants (24 times). The emotion word *enthusiastic* was also used 21 times for this. Both *enthusiastic* and *confident* have similar valence levels (from Figure 1), which could be the cause of confusion. Moreover, this confusion between *confident* and *enthusiastic* may not be detrimental to the experience of the users of a healthcare robot speaking with these emotions.

The free-response test was conducted after the forced-response test. The effect of this prior knowledge of emotions’ names was evident in the responses they provided. It could be expected that the participants may have given more common emotion words such as *happy*, *angry* or *sad* if the free-response test had been conducted before the forced-response test. However, this ordering was deliberately done to familiarise the participants with the names of the secondary emotions.

### 5.3. Task III-Naturalness Rating on Five-Point Scale

The overarching extension of this research aims to synthesise emotional voices for healthcare robots. Emotional speech synthesis aims to create emotional voices that are similar to how humans portray emotions. The naturalness of the emotional voice developed here was evaluated subjectively. In this task, the participants listened to a synthesised emotional sentence and rated the perceived level of naturalness. The question asked to the participants was “Rate the naturalness of this voice (by naturalness, we mean how close this voice is to the human voice) with five being the most natural” on a discrete scale of one to five. The five levels were based on the levels of naturalness defined in [52], which were very unnatural, unnatural, neutral, natural and very natural. The sentences used were a subset of the sentences used for the forced-response test in Task I. For each secondary emotion, two sentences were evaluated by 29 participants, giving a total of 290 evaluations.

**Figure 8:**
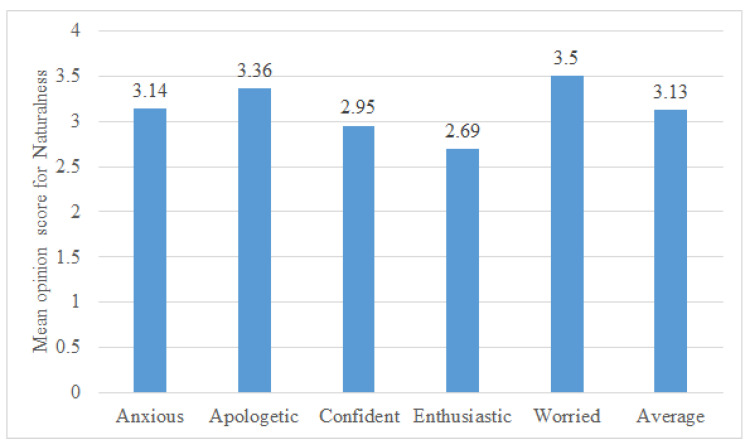
Naturalness rating boxplots for synthesised secondary emotional speech in percentage.

Figure 8 shows the mean opinion score of naturalness. The average score across all five emotions is also included. The perception of *enthusiastic* was found to be the least natural. However, all emotions’ naturalness rating was greater than 2.5, which indicated that the voice was not perceived as unnatural (based on the five naturalness levels described in [52]). The fact that even though the emotions were modelled on *synthesised speech*, the participants still felt that the emotional sentences were close to having a natural quality is a positive result.

### 5.4. Task IV-Comfort Level Rating on Five-Point Scale

As the emotional speech developed here is for healthcare robots, the people listening to the speech from the robots have to find it comfortable to listen to. In this task, the participants listened to a synthesised emotional sentence and rated their perceived comfort level. The question asked to the participants was “Rate your comfort level in listening to this voice for a long time (By comfort level, we mean if you can listen to this voice for a long time-more than 1 min) with five being most comfortable”. The comfort level was the ease of listening, defined by [52] as the ease of listening to the voice for long periods of time. The participants had to rate the voice on a five-point discrete scale, with five being the most comfortable. These five levels were based on the levels defined by [52] as very difficult, difficult, neutral, easy and very easy, with discrete levels varying from one to five. For each secondary emotion, one sentence was evaluated by 29 participants, giving a total of 145 evaluations. The sentences used for this section were a subset of the collection of sentences used in Task I.

**Figure 9:**
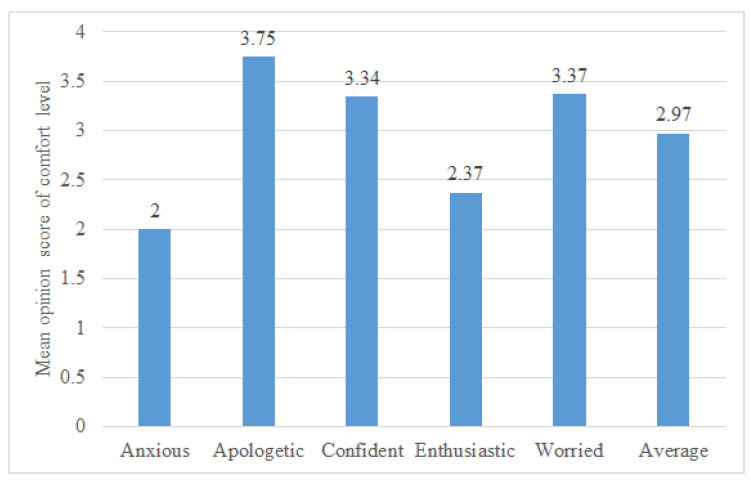
Comfort level rating boxplots for synthesised secondary emotional speech in percentage.

Figure 9 shows the mean opinion score of the comfort level perception. The average score across all five emotions is also included. All the emotions had a mean opinion score above two on the comfort level five-point scale, which indicated it was neutral, easy or very easy to listen to [52]. The perception of *anxious* was found to have the least comfort rating among all five emotions evaluated. For the emotional text-to-speech synthesis developed here, the voice synthesised here was expected to be suitable as, on average, participants found it comfortable to listen to (mean opinion score of 2.97).

## 6. Discussion

In this paper, the focus was on developing a low-resource approach for emotional speech synthesis of five secondary emotions by modelling prosody features. The three prosody features have been well-studied and used for the synthesis of primary emotions and speaking styles [10,13,15,20]. Here, we analysed the impact of secondary emotions on these three prosody features. The acoustic analysis results showed that these three prosody features were impacted by secondary emotions. This was the motivation to develop a proof-of-concept emotional TTS synthesis system that synthesised secondary emotions by modelling the three prosody features only. Subjective tests provided a strong indication that the approach of modelling these prosody features was promising for secondary emotions synthesis, at least for the five secondary emotions considered here. This justifies future work in which we will pursue a deeper investigation of incorporating other acoustic features for synthesis. Such an investigation is essential for the emotion pairs that were found to be the most confusing in the perception tests such as *apologetic-worried.*

Instead of using the qualitative statistics of $f_{0}$, we focused on the $f_{0}$ contour modelling. Contour-level modelling of $f_{0}$ was used for emotional speech synthesis in a study [15] based on the ToBI model (a qualitative model). We expected the subtleties in the $f_{0}$ contour could be picked up by quantitative modelling rather than a qualitative approach. Moreover, by using the ToBI model, one can only get tags for the various tones and breaks, and then the $f_{0}$ values would have to be calculated by other approaches. The Fujisaki model, on the other hand, provides an equation for the $f_{0}$ contour, thereby facilitating resynthesis. Moreover, this approach seemed to be picking up the subtle changes in the $f_{0}$ contour introduced by the secondary emotions (as seen in the results in Section 3.2). Hence, in this investigation, we utilised the traditional handcrafted feature extraction approach and married it with modern machine learning to effectively synthesise secondary emotions. There was an emphasis on how the $f_{0}$ contour data were prepared for the modelling. This involved parameterising the $f_{0}$ contour using the Fujisaki model and hand-correction-a task requiring phonetic knowledge about accents and phrases.

Most emotional synthesisers use quantitative models such as mean or range variations on prosody features to synthesise emotional speech [13,20], or they rely on spectrogram-based features and use deep learning models to learn patterns for speech synthesis [11,18]. The former statistical-features-based model can miss the subtleties introduced in the $f_{0}$ contour by the secondary emotions and the latter relies on a large database, which is not easy to develop for all emotions and all languages. The approach presented in this study utilised the strength of speech signal processing models to develop a low-resource emotional speech synthesiser. This approach can be easily trained for other emotions as well, even if a small database of the emotion is available.

The secondary emotions studied here are novel to emotional speech synthesis research, even though they are commonly used in human conversations. It was found that all three prosody features were arousal-differentiating features. However, valence-differentiating features may be particularly crucial for secondary emotions, as the arousal level differences for these nuanced emotions were not as dominant as the primary emotions. For example, for secondary emotions, the arousal difference between *worried*, *confident* and *enthusiastic* was not much, and to differentiate them, valence-level features may also be needed. This will be a focus of future research. The subjective test results showed that at least three out of the five emotions could be adequately modelled by this approach. This can be seen in the confusion matrix in Table 4 and in Table 5, where the emotions *anxious*, *enthusiastic* and *confident* could be perceived well by the participants. However, the emotions *apologetic* and *worried* seemed to be confused with one another and were not very well recognised in the free-response test either. These emotions will have to be studied in detail to model them better.

The parametric modelling of the $f_{0}$ contour was a crucial addition. This contributed to a low-resource approach to emotional speech synthesis. This can be further expanded by collecting small databases for other secondary emotions and creating models for them. Newer studies have used sequence-to-sequence modelling [51] for predicting the $f_{0}$ contour. Rather than a direct prediction of the contour, a prediction of the $f_{0}$ contour’s Fujisaki model parameters using a sequence-to-sequence framework may be a better approach that facilities resynthesis. However, such a neural-network-based approach may require a larger database, and further experimentation needs to be done on the feasibility of the approach. Speech research initially tried to model the human speech production system. With emerging trends to employ deep learning in speech technology research, all the features are extracted by automatic processes and fed into a “black box”, thus often lacking an understanding of the acoustic features impacted by emotions. This research, on the other hand, attempted to understand three prosody features and their impact on secondary emotions, which were used to synthesise five secondary emotions.

## 7. Conclusions

This paper addressed two research questions - can prosody features model secondary emotions, and how to create a low-resource emotional text to speech synthesis system for secondary these emotions. It was found that the $f_{0}$ contour, speech rate and mean intensity were impacted by the five secondary emotions. Based on a statistical analysis, the $f_{0}$ contour was modelled using a quantitative model called the Fujisaki model, and the other two features were modelled by rules. To answer the second research question, a TTS synthesis system for secondary emotions was developed. A transformation model was then developed to transform the $f_{0}$ contour’s Fujisaki parameters to those of emotional speech. The features used for the transformation were the input text and the emotions tag. After the transformation, the speech signal was resynthesised to produce the intended emotion. Moreover, a detailed subjective test was conducted to evaluate the performance of the emotional speech synthesis system.

What makes this study different from past studies is the attempt to synthesise less- explored secondary emotions. Modelling the $f_{0}$ contour quantitatively instead of only using qualitative measures could capture the subtle changes in the $f_{0}$ contour due to the secondary emotions, and also facilitate direct resynthesis, while for modelling the stronger primary emotions, the mean and range features may be sufficient. However, for the secondary emotions, changes in the accent and phrase levels have to be captured.

The development of larger databases with variations such as emotions, speakers and linguistic contexts can produce a more robust emotion transformation model. Such a database may be expensive and difficult to obtain. The focus on understanding the properties of the speech signal and what features differentiate one emotion from another is the approach followed here. This will be beneficial in cases similar to this study, where the database is not large enough to perform advanced machine-learning-based modelling. Only three prosody features were modelled here. This modelling produced above-chance results in correctly identifying the secondary emotions. Further research can look at how other acoustic features, such as spectral and glottal features, can be modelled and incorporated into an emotional speech synthesis system for secondary emotions. In the future, an emotion-based linguistic analysis of the input text will be advantageous for more accurate predictions of accent and phrase commands.

## Figures and Tables

**Table 2:** Features used for $f_{0}$ contour transformation.

Feature	Description	Extraction Method
Linguistic context features	Count = 102, e.g., accented/unaccented, vowel/consonant	Text analysis at the phonetic level using MaryTTS.
Nonemotional $f_{0}$ contour Fujisaki model parameters	Five Fujisaki parameters-$A_{pN}$, $A_{aN}$, $Dur\_phr_{N}$, $Dur\_acc_{N}$, $F_{bN}$	Passing nonemotional speech to AutoFuji extractor.
Emotion tag	Five primary and five secondary emotions	Each emotion tag is assigned to the sentence
Speaker tag	Two male speakers	Speaker tag is assigned

**Table 3:** Mean speech rate value for five secondary emotions.

Secondary Emotion	Mean Speech Rate (Syllables/s)	Mean Intensity (dB)
*anxious*	3.25	58.24
*apologetic*	2.93	55.14
*confident*	3.20	59.50
*enthusiastic*	3.24	63.91
*worried*	2.99	56.34

**Table 4:** Hit rates from forced-response test (ANX: anxious, APO: apologetic, CONF: confident, ENTH: enthusiastic, WOR: worried).

	APO	ANX		APO	ENTH
APO	**97.9 %**	2.1%	APO	**100%**	0%
ANX	0%	**100%**	ENTH	1.4%	**98.6%**
	CONF	ANX		APO	WOR
CONF	**88.3%**	11.7%	APO	**64.3%**	35.7%
ANX	12.4%	**87.6%**	WOR	32.4%	**67.6%**
	ENTH	ANX		CONF	ENTH
ENTH	**78.6%**	21.4%	CONF	**69%**	31%
ANX	24.8%	**75.2%**	ENTH	30.3%	**69.7%**
	WOR	ANX		CONF	WOR
WOR	**97.9%**	2.1%	CONF	**95.2%**	4.8%
ANX	4.1%	**95.9%**	WOR	22.8%	**77.2%**
	APO	CONF		WOR	ENTH
APO	**94.5%**	5.5%	WOR	**97.9%**	2.1%
CONF	9.7%	**90.3%**	ENTH	0.7%	**99.3%**

**Table 5:** Perceived emotion words in free-response test (times used).

Actual Emotions	Emotion Words by Participants (Count of Times Used)
**Anxious**	**Anxious (41)**, enthusiastic (9), neutral (4), confident (3), energetic (1)
**Apologetic**	**Apologetic (35)**, worried (22), worried/sad (1)
**Confident**	**Confident (34)**, enthusiastic (9), worried (8), neutral (5), authoritative (1), demanding (1)
**Enthusiastic**	Confident (24), **enthusiastic (21)**, neutral (4), apologetic (3), worried (5), encouraging (1)
**Worried**	**Worried (38)**, apologetic (12), anxious (5), condescending (1), confident (1), neutral (1)

## Data Availability

No new data were created or analyzed in this study. Data sharing is not applicable to this article.

## References

[1] Eyssel F., Ruiter L.D., Kuchenbrandt D., Bobinger S., Hegel F. ‘If you sound like me, you must be more human’: On the interplay of robot and user features on human-robot acceptance and anthropomorphism. Proceedings of the International Conference on Human-Robot Interaction.

[2] James J., Watson C.I., MacDonald B. Artificial empathy in social robots: An analysis of emotions in speech. Proceedings of the IEEE International Symposium on Robot & Human Interactive Communication.

[3] James J., Balamurali B., Watson C.I., MacDonald B. (2020). Empathetic Speech Synthesis and Testing for Healthcare Robots. Int. J. Soc. Robot..

[4] Ekman P. (1992). An argument for basic emotions. Cogn. Emot..

[5] Schröder M. Emotional Speech Synthesis: A Review. Proceedings of the Eurospeech.

[6] Becker-Asano C., Wachsmuth I. (2008). Affect Simulation with Primary and Secondary Emotions. Proceedings of the Intelligent Virtual Agents.

[7] Damasio A. (1994). Descartes’ Error, Emotion Reason and the Human Brain.

[8] James J., Watson C., Stoakes H. Influence of Prosodic features and semantics on secondary emotion production and perception. Proceedings of the International Congress of Phonetic Sciences.

[9] Murray I.R., Arnott J.L. (1995). Implementation and testing of a system for producing emotion-by-rule in synthetic speech. Speech Commun..

[10] Tao J., Kang Y., Li A. (2006). Prosody conversion from neutral speech to emotional speech. IEEE Trans. Audio Speech Lang. Process..

[11] Skerry-Ryan R., Battenberg E., Xiao Y., Wang Y., Stanton D., Shor J., Weiss R.J., Clark R., Saurous R.A. Towards end-to-end prosody transfer for expressive speech synthesis with tacotron. Proceedings of the International Conference on Machine Learning.

[12] An S., Ling Z., Dai L. Emotional statistical parametric speech synthesis using LSTM-RNNs. Proceedings of the APSIPA Conference.

[13] Vroomen J., Collier R., Mozziconacci S. Duration and intonation in emotional speech. Proceedings of the Third European Conference on Speech Communication and Technology.

[14] Masuko T., Kobayashi T., Miyanaga K. A style control technique for HMM-based speech synthesis. Proceedings of the International Conference on Spoken Language Processing.

[15] Pitrelli J.F., Bakis R., Eide E.M., Fernandez R., Hamza W., Picheny M.A. (2006). The IBM expressive text-to-speech synthesis system for American English. IEEE Trans. Audio Speech Lang. Process..

[16] Yamagishi J., Kobayashi T., Tachibana M., Ogata K., Nakano Y. Model adaptation approach to speech synthesis with diverse voices and styles. Proceedings of the International Conference on Acoustics, Speech and Signal Processing.

[17] Barra-Chicote R., Yamagishi J., King S., Montero J.M., Macias-Guarasa J. (2010). Analysis of statistical parametric and unit selection speech synthesis systems applied to emotional speech. Speech Commun..

[18] Tits N. A Methodology for Controlling the Emotional Expressiveness in Synthetic Speech-a Deep Learning approach. Proceedings of the International Conference on Affective Computing and Intelligent Interaction.

[19] Zhang M., Tao J., Jia H., Wang X. Improving HMM based speech synthesis by reducing over-smoothing problems. Proceedings of the International Symposium on Chinese Spoken Language Processing.

[20] Murray I., Arnott J.L. (1993). Toward the simulation of emotion in synthetic speech: A review of the literature on human vocal emotion. J. Acoust. Soc. Am..

[21] James J., Tian L., Watson C. An open source emotional speech corpus for human robot interaction applications. Proceedings of the Interspeech.

[22] Scherer K. (2005). What are emotions? And how can they be measured?. Soc. Sci. Inf..

[23] Paltoglou G., Thelwall M. (2013). Seeing Stars of Valence and Arousal in Blog Posts. IEEE Trans. Affect. Comput..

[24] Sitaula C., He J., Priyadarshi A., Tracy M., Kavehei O., Hinder M., Withana A., McEwan A., Marzbanrad F. (2022). Neonatal Bowel Sound Detection Using Convolutional Neural Network and Laplace Hidden Semi-Markov Model. IEEE/ACM Trans. Audio Speech Lang. Process..

[25] Burne L., Sitaula C., Priyadarshi A., Tracy M., Kavehei O., Hinder M., Withana A., McEwan A., Marzbanrad F. (2022). Ensemble Approach on Deep and Handcrafted Features for Neonatal Bowel Sound Detection. IEEE J. Biomed. Health Inform..

[26] Winkelmann R., Harrington J.J.K. (2017). EMU-SDMS: Advanced speech database management and analysis in R. Comput. Speech Lang..

[27] R Core Team (2017). R: A Language and Environment for Statistical Computing.

[28] Wickham H. (2016). ggplot2: Elegant Graphics for Data Analysis.

[29] James J., Mixdorff H., Watson C. Quantitative model-based analysis of *F*_0_ contours of emotional speech. Proceedings of the International Congress of Phonetic Sciences.

[30] Hui C.T.J., Chin T.J., Watson C. Automatic detection of speech truncation and speech rate. Proceedings of the SST.

[31] Hirose K., Fujisaki H., Yamaguchi M. Synthesis by rule of voice fundamental frequency contours of spoken Japanese from linguistic information. Proceedings of the IEEE International Conference on Acoustics, Speech, and Signal Processing.

[32] Nguyen D.T., Luong M.C., Vu B.K., Mixdorff H., Ngo H.H. Fujisaki Model based f0 contours in Vietnamese TTS. Proceedings of the International Conference on Spoken Language Processing.

[33] Gu W., Lee T. Quantitative analysis of f0 contours of emotional speech of Mandarin. Proceedings of the 8th ISCA Spee Synthesis Workshop.

[34] Amir N., Mixdorff H., Amir O., Rochman D., Diamond G.M., Pfitzinger H.R., Levi-Isserlish T., Abramson S. Unresolved anger: Prosodic analysis and classification of speech from a therapeutic setting. Proceedings of the Speech Prosody.

[35] Mixdorff H., Cossio-Mercado C., Hönemann A., Gurlekian J., Evin D., Torres H. Acoustic correlates of perceived syllable prominence in German. Proceedings of the Annual Conference of the International Speech Communication Association.

[36] Boersma P., Weenink D. (2019). Praat: Doing Phonetics by Computer [Computer Program]. Version 6.0.46. https://www.fon.hum.uva.nl/praat/.

[37] Mixdorff H. A novel approach to the fully automatic extraction of Fujisaki model parameters. Proceedings of the IEEE Int. Conf. on Acoustics, Speech, and Signal Processing.

[38] Mixdorff H., Fujisaki H. A quantitative description of German prosody offering symbolic labels as a by-product. Proceedings of the International Conference on Spoken Language Processing.

[39] Watson C.I., Marchi A. Resources created for building New Zealand English voices. Proceedings of the Australasian International Conference of Speech Science and Technology.

[40] Jain S. (2015). Towards the Creation of Customised Synthetic Voices using Hidden Markov Models on a Healthcare Robot. Master’s Thesis.

[41] Schröder M., Trouvain J. (2003). The German text-to-speech synthesis system MARY: A tool for research, development and teaching. Int. J. Speech Technol..

[42] Boersma P. (1993). Accurate short-term analysis of the fundamental frequency and the harmonics-to-noise ratio of a sampled sound. Inst. Phon. Sci..

[43] Kisler T., Schiel F., Sloetjes H. Signal processing via web services: The use case WebMAUS. Proceedings of the Digital Humanities Conference.

[44] Liaw A., Wiener M. (2002). Classification and Regression by Random Forest. R News 2.3.

[45] Yoav F., Robert E S. Experiments with a new boosting algorithm. Proceedings of the International Conference on Machine Learning.

[46] Pedregosa F., Varoquaux G., Gramfort A., Michel V., Thirion B., Grisel O., Blondel M., Prettenhofer P., Weiss R., Dubourg V. (2011). Scikit-learn: Machine Learning in Python. J. Mach. Learn. Res..

[47] Mendes-Moreira J., Soares C., Jorge A.M., Sousa J.F.D. (2012). Ensemble approaches for regression: A survey. ACM Comput. Surv..

[48] Eide E., Aaron A., Bakis R., Hamza W., Picheny M., Pitrelli J. A corpus-based approach to expressive speech synthesis. Proceedings of the ISCA ITRW on Speech Synthesis.

[49] Ming H., Huang D.Y., Dong M., Li H., Xie L., Zhang S. Fundamental Frequency Modeling Using Wavelets for Emotional Voice Conversion. Proceedings of the International Conference on Affective Computing and Intelligent Interaction.

[50] Lu X., Pan T. Research On Prosody Conversion of Affective Speech Based on LIBSVM and PAD Three Dimensional Emotion Model. Proceedings of the Wkhp on Advanced Research & Tech in Industry Applications.

[51] Robinson C., Obin N., Roebel A. Sequence-To-Sequence Modelling of *F*_0_ for Speech Emotion Conversion. Proceedings of the International Conference on Acoustics, Speech, and Signal Processing.

[52] Viswanathan M., Viswanathan M. (2005). Measuring speech quality for text-to-speech systems: Development and assessment of a modified mean opinion score (MOS) scale. Comput. Speech Lang..

